# Synthesis of New *β*-Lactams Bearing the Biologically Important Morpholine Ring and POM Analyses of Their Antimicrobial and Antimalarial Activities

**Published:** 2019

**Authors:** Aliasghar Jarrahpour, Roghayeh Heiran, Véronique Sinou, Christine Latour, Lamia Djouhri Bouktab, Jean Michel Brunel, Javed Sheikh, Taibi Ben Hadda

**Affiliations:** a *Department of Chemistry, College of Sciences, Shiraz University, Shiraz, Iran. *; b *Department of Chemistry, Estahban Higher Education Center, Estahban, Iran.*; c *UMR-MD3 Relation hoˆte-parasites, Physiopathologie and Pharmacologie, Faculte´ de Pharmacie, Aix-Marseille Universite´, Bd Jean Moulin, Marseille, 13385, France.*; d *Centre de Recherche en Cance´rologie de Marseille (CRCM), UM 105, CNRS, UMR7258, Institut Paoli Calmettes, Inserm, U1068, Faculte´ de Pharmacie, Aix-Marseille Universite´, Bd Jean Moulin, Marseille, 13385, France. *; e *Department of Chemistry, Dhote Bandhu Science College, Gondia, 441614 India.*; f *Laboratoire de Chimie des Matériaux (LCM), Département de Chimie, Facultés des Sciences, Université Mohammed Premier, Oujda, 60000, Morocco.*

**Keywords:** *β-*Lactam, 2-Azetidinones, Acylation, Antimalarial activities, POM analyses

## Abstract

Some new *β-*lactams bearing biologically important morpholine ring have been synthesized by acylation of amino *β-*lactams in the presence of morpholine-4-carbonyl chloride. These novel *β*-lactams were prepared under mild reaction conditions without any solvent in short reaction times. Their biological activities have been examined against microbial agents such as *Staphylococcus aureus* (*S. aureus*)*, Escherichia coli *(*E. coli*), *Pseudomonas aeruginosa* (*P. aeruginosa*) and fungi such as *Candida albicans* (*C. albicans*) and *Candida glabrata* (*C. glabrata*). They have been also tested against *Plasmodium falciparum* K14 resistant strain and showed moderate to good IC_50_ values.

## Introduction

Chemists have always been interested in the synthesis of heterocyclic compounds because of their important biological activities. *b-*Lactam derivatives, characterized by the presence of an azetidine-2-one ring (1) have shown various medical applications, due to their low toxicity (2). This class of drugs is still in extensive use (3) and can be classified into antimalarial (4, 5), antimicrobial and antifungal (6), antitubercular (7), antidiabetic (8), anticancer (9), antiparkinsonian (10), antitumor (11) and anti-HIV (12) agents. Anyway, the wide use of these agents have caused the bacteria to produce different type of *β*-lactamases (*β*-Lases) (13)^ .^So, the phenomenon of bacterial resistance stimulates a lot of work being devoted to novel 2-azetidinone derivatives with modification of structure of known active compounds (14).

**Figure 1:**
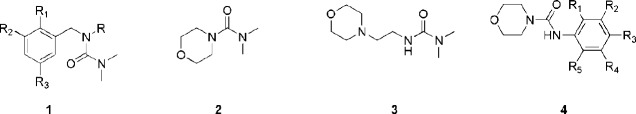
Representative examples of some biologically active urea-like compounds

Urea derivatives have got an important role in modern medicinal chemistry because of their biological activities (15) such as enzyme inhibitors (16), CXCR3 antagonist (17), anticonvulsant (18), colchicine-blinding antagonist (19), and anticancer activities (20). Zhu *et al.* synthesized some urea-like compounds such as **1 **(21),** 2** and **3 **(Figure 1) (22). It was found that incorporating a heterocycle, especially a morpholine ring exhibited remarkable activity toward human cancer cell lines. Although there is less report about antifungal and antibacterial activities of urea (23), recently a series of *N*-alkyl substituted urea derivatives **4 **(Figure 1) (24) have been synthesized. In general, the compounds bearing morpholine moiety, exhibited better antibacterial and antifungal activities. In this study we report the synthesis of some new modified *β*-lactams bearing the biologically important morpholine ring under solvent free conditions as well as their potent antimicrobial and antimalarial activities.

## Experimental

*General*

All required chemicals were purchased from the Merck, and Acros chemical companies. CH_2_Cl_2_ and Et_3_N were dried by distillation over CaH_2_ and then stored over 4Å molecular sieves. ^1^H NMR and ^13^C NMR spectra were recorded in DMSO-d_6_ using a Bruker Avance DPX instrument (operating at 250 MHz for ^1^H NMR and 62.9 MHz for ^13^C NMR). Chemical shifts were reported in ppm (δ) downfield from TMS. All of the coupling constants (*J*) are in Hertz. IR Spectra were run on a Shimadzu FT-IR 8300 spectrophotometer. Elemental analyses were run on a Thermo Finnigan Flash EA-1112 series. Melting points were determined in open capillaries with a thermo scientific melting point apparatus. Thin-layer chromatography (TLC) was carried out on silica gel 254 analytical sheets obtained from Fluka.

*General procedure for the synthesis of compounds (* ***2a-n)***

A mixture of *β*-lactams bearing amino moiety **1a-n** (0.50 mmol) prepared according to our previous report (25), morpholine-4-carbonyl chloride (1.00 mmol), and Et_3_N (0.75 mmol) were mixed in a mortar and ground with a pastle at room temperature for 1.5 h to produce new *β*-lactams **2a-n.** The progress of the reaction was monitored by TLC. The crude product was puriﬁed by thick layer chromatography (eluent 4:1 CHCl_3_/EtOAc) to yield pure *β*-lactams **2a-n.**

*4-(4-Aminophenyl)-1-(4-methoxyphenyl)-3-(naphthalen-2-yloxy) - azetidine-2-one* ***(1d)***

White crystals, yield 65%, mp: 150-152 °C; IR (KBr, cm^−1^): 1730 (CO, *β*-lactam), 3388, 3475 (NH_2_). ^1^H-NMR (DMSO-d_6_) δ (ppm): 3.68 (OMe, s, 3H), 5.01 (NH_2_, s, 2H), 5.56 (H-4, d, *J* = 4.6 Hz, 1H), 5.85 (H-3, d, *J* = 4.6 Hz, 1H), 6.35 (ArH, d, *J* = 8.4 Hz, 2H), 6.88–7.78 (ArH, m, 13H). ^13^C-NMR (DMSO-d_6_) δ (ppm): 55.2 (OMe), 61.1 (C-4), 80.5 (C-3), 198.6, 113.4, 114.4, 117.9, 118.5, 119.1, 123.9, 126.4, 126.7, 127.5, 128.8, 128.9, 129.2, 130.3, 133.7, 148.6, 154.3, 155, 8 (aromatic carbons), 162.1 (CO, *β*-lactam). Anal. Calcd. for C_26_H_22_N_2_O_3_: C, 76.08; H, 5.40; N, 6.82. Found: C, 76.19; H, 5.63; N, 6.87%.

*4-(4-Aminophenyl)-3-(4-chlorophenoxy)-1-(4-ethoxyphenyl)-azetidine-2-one* ***(1e)***

White solid, yield 57%, mp: 164-168 °C; IR (KBr, cm^−1^): 1743 (CO, *β*-lactam), 3379, 3479 (NH_2_). ^1^H-NMR (DMSO-d_6_) δ (ppm): 1.26 (Me, t, *J* = 7.0 Hz, 3H), 3.92 (OCH_2_, q, *J* = 7.0 Hz, 2H), 5.06 (NH_2_, s, 2H), 5.53 (H-4, d, *J* = 4.6 Hz, 1H), 5.69 (H-3, d, *J* = 4.6 Hz, 1H), 6.36 (ArH, d, *J* = 8.4 Hz, 2H), 6.79 (ArH, d, *J* = 9.0 Hz, 2H), 6.84 (ArH, d, *J* = 9.0 Hz, 2H), 6.95 (ArH, d, *J* = 8.4 Hz, 2H), 7.17 (ArH, d, *J* = 9.0 Hz, 2H), 7.18 (ArH, d, *J* = 9.0 Hz, 2H). ^13^C-NMR (DMSO-d_6_) δ (ppm): 14.5 (Me), 60.9 (OCH_2_), 63.1 (C-4), 80.4 (C-3), 113.4, 114.9, 116.7, 118.5, 118.9, 125.2, 128.9, 130.1, 148.7, 155.1, 155.3 (aromatic carbons), 161.8 (CO, *β*-lactam). Anal. Calcd. for C_23_H_21_ClN_2_O_3_: C, 67.56; H, 5.18; N, 6.85. Found: C, 67.47; H, 5.20; N, 6.78%.

*4-(4-Aminophenyl)-3-(2,4-dichlorophenoxy)-1-(4-ethoxyphenyl) azetidine-2-one* ***(1f)***

White solid, yield 71%, mp: 196-198 °C; IR (KBr, cm^−1^): 1759 (CO, *β*-lactam), 3350, 3475 (NH_2_). ^1^H-NMR (DMSO-d_6_) δ (ppm): 1.26 (Me, t, *J* = 6.9 Hz, 3H), 3.93 (OCH_2_, q, *J* = 6.9 Hz, 2H), 5.07 (NH_2_, s, 2H), 5.45 (H-4, d, *J* = 4.5 Hz, 1H), 5.82 (H-3, d, *J* = 4.5 Hz, 1H), 6.36 (ArH, d, *J* = 8.4 Hz, 2H), 6.85 (ArH, d, *J* = 8.9 Hz, 2H), 6.97 (ArH, d, *J* = 8.4 Hz, 2H), 7.05 (ArH, d, *J* = 8.9 Hz, 1H), 7.19–7.28 (ArH, m, 3H), 7.41 (ArH, s, 1H). ^13^C-NMR (DMSO-d_6_) δ (ppm): 14.5 (Me), 60.6 (OCH_2_), 63.1 (C-4), 80.5 (C-3), 113.4, 114.9, 115.8, 118.5, 122.0, 125.4, 127.6, 128.9, 129.2, 130.0, 148.8, 150.8, 155.1 (aromatic carbons), 161.2 (CO, *β*-lactam). Anal. Calcd. for C_23_H_20_Cl_2_N_2_O_3_: C, 62.31; H, 4.55; N, 6.32. Found: C, 62.46; H, 4.69; N, 6.31%.

*4-(4-Aminophenyl)-1-(4-ethoxyphenyl)-3-phenoxy azetidin-2-one* ***(1g)***

White solid, yield 87%, mp: 182-184 °C; IR (KBr, cm^−1^): 1736 (CO, *β*-lactam), 3350, 3446 (NH_2_). ^1^H-NMR (DMSO-d_6_) δ (ppm): 1.28 (Me, t, *J* = 6.8 Hz, 3H), 3.93 (OCH_2_, q, *J* = 6.8 Hz, 2H), 5.05 (NH_2_, s, 2H), 5.43 (H-4, d, *J* = 4.4 Hz, 1H), 5.68 (H-3, d, *J* = 4.4 Hz, 1H), 6.38 (ArH, d, *J* = 8.0 Hz, 2H), 6.78–6.87 (ArH, m, 5H), 6.98 (ArH, d, *J* = 8.0 Hz, 2H), 7.11–7.23 (ArH, m, 4H). ^13^C-NMR (DMSO-d_6_) δ (ppm): 14.5 (Me), 61.1 (OCH_2_), 63.1 (C-4), 80.4 (C-3), 113.4, 114.9, 115.0, 118.5, 119.2, 121.5, 128.9, 129.2, 130.2, 148.7, 155.0, 156.6 (aromatic carbons), 162.2 (CO, *β*-lactam). Anal. Calcd. for C_23_H_22_N_2_O_3_: C, 73.78; H, 5.92; N, 7.48. Found: C, 73.86; H, 6.03; N, 7.35%.

*4-(4-Aminophenyl)-1-(4-ethoxyphenyl)-3-(naphthalen-2-yloxy) azetidine-2-one* ***(1h)***

Light brown solid, yield 94%, mp: 160-162 °C; IR (KBr, cm^−1^): 1735 (CO, *β*-lactam), 3379, 3465 (NH_2_). ^1^H-NMR (DMSO-d_6_) δ (ppm): 1.23 (Me, t, *J* = 7.0 Hz, 3H), 3.93 (OCH_2_, q, *J* = 7.0 Hz, 2H), 4.99 (NH_2_, s, 2H), 5.55 (H-4, d, *J* = 4.6 Hz, 1H), 5.83 (H-3, d, *J* = 4.6 Hz, 1H), 6.34 (ArH, d, *J* = 8.4 Hz, 2H), 6.86 (ArH, d, *J* = 9.0 Hz, 2H), 6.96–7.32 (ArH, m, 8H), 7.67 (ArH, d, *J* = 9.0 Hz, 1H), 7.74 (ArH, d, *J* = 9.0 Hz, 2H). ^13^C-NMR (DMSO-d_6_) δ (ppm): 14.5 (Me), 61.1 (OCH_2_), 63.1 (C-4), 80.4 (C-3), 108.5, 113.4, 114.9, 117.9, 118.5, 119.1, 123.9, 126.4, 126.7, 127.5, 128.8, 128.9, 129.2, 130.2, 133.7, 148.6, 154.3, 155.1 (aromatic carbons), 162.1 (CO, *β*-lactam). Anal. Calcd. for C_27_H_24_N_2_O_3_: C, 76.39; H, 5.70; N, 6.60. Found: C, 76.35; H, 5.84; N, 6.63%.

*4-(4-Aminophenyl)-3-(4-chlorophenoxy)-1-(4-methoxybenzyl) azetidine-2-one* ***(1i)***

Cream solid, yield 75%, mp: 160-162 °C; IR (KBr, cm^−1^): 1749 (CO, *β*-lactam), 3379, 3456 (NH_2_). ^1^H-NMR (DMSO-d_6_) δ (ppm): 3.71 (OMe, s, 3H), 3.72 (CH_2_, d, *J *= 15.1, 1H), 4.57 (CH_2_, d, *J *= 15.1, 1H), 4.77 (H-4, d, *J* = 4.2 Hz, 1H), 5.06 (NH_2_, s, 2H), 5.52 (H-3, d, *J* = 4.2 Hz, 1H), 6.38 (ArH, d, *J* = 8.4 Hz, 2H), 6.75 (ArH, d, *J* = 9.0 Hz, 2H), 6.84 (ArH, d, *J* = 8.4 Hz, 2H), 6.85 (ArH, d, *J* = 8.7 Hz, 2H), 7.06 (ArH, d, *J* = 8.7 Hz, 2H), 7.12 (ArH, d, *J* = 9.0 Hz, 2H). ^13^C-NMR (DMSO-d_6_) δ (ppm): 42.5 (CH_2_), 55.0 (OMe) 60.4 (C-4), 81.3 (C-3), 113.4, 113.9, 116.6, 118.9, 125.0, 127.3, 128.9, 129.1, 129.3, 148.6, 155.3, 158.6 (aromatic carbons), 164.5 (CO, *β*-lactam). Anal. Calcd. for C_23_H_21_ClN_2_O_3_: C, 67.56; H, 5.18; N, 6.85. Found: C, 67.78; H, 5.25; N, 7.00%.

*4-(3-Aminophenyl)-3-(4-chlorophenoxy)-1-(4-methoxy phenyl) azetidine-2-one* ***(1k)***

White solid, yield 95%, mp: 174-176 °C; IR (KBr, cm^−1^): 1747 (CO, *β*-lactam), 3369, 3437 (NH_2_). ^1^H-NMR (DMSO-d_6_) δ (ppm): 3.68 (OMe, s, 3H), 5.04 (NH_2_, s, 2H), 5.18 (H-4, d, *J* = 4.7 Hz, 1H), 5.39 (H-3, d, *J* = 4.7 Hz, 1H), 6.64–7.00 (ArH, m, 8H), 7.20 (ArH, d, *J* = 8.9 Hz, 4H). ^13^C-NMR (DMSO-d_6_) δ (ppm): 55.2 (OMe), 61.1 (C-4), 80.6 (C-3), 112.7, 114.0, 114.5, 115.8, 116.9, 118.4, 125.4, 128.6, 129.0, 129.3, 133.4, 148.4, 155.5, 155.9 (aromatic carbons), 161.8 (CO, *β*-lactam). Anal. Calcd. for C_22_H_19_ClN_2_O_3_: C, 66.92; H, 4.85; N, 7.09. Found: C, 66.85; H, 4.64; N, 7.16%.

*N-(4-(1-(4-methoxyphenyl)-4-oxo-3-phenoxyazetidin-2-yl)phenyl)morpholine-4 carboxamide* ***(2a)***

White powder, yield 88%, mp: 194-196 °C; IR (KBr, cm^-1^): 1666 (CO, urea), 1739 (CO, *β*-lactam), 3458 (NH). ^1^H NMR (DMSO-d_6_) δ (ppm): 3.35 (m, 4H, CH_2_-N), 3.52 (m, 4H, CH_2_-O), 3.67 (s, 3H, OMe), 5.58 (d, *J *4.6 Hz, 1H, H-4), 5.76 (d, *J* 4.6 Hz, 1H, H-3), 6.79–7.36 (m, 13H, ArH), 8.51 (s, 1H, NH, D_2_O exchange). ^13^C NMR (62.9 MHz, DMSO-d_6_) δ (ppm): 44.1 (CH_2_-N), 55.2 (OMe), 60.7 (C-4), 65.9 (CH_2_-O), 80.5 (C-3), 114.5, 114.9, 118.5, 119.1, 121.7, 126.0, 128.2, 129.3, 130.1, 140.4, 155.8, 156.5 (aromatic carbons), 154.9 (CO, urea), 162.1 (CO, *β*-lactam); Anal. calcd for C_27_H_27_N_3_O_5_ (473.52): C, 68.48; H, 5.75; N, 8.87%. Found: C, 68.44; H, 5.77; N 8.85%. 

*N-(4-(3-(2,4-dichlorophenoxy)-1-(4-methoxyphenyl)-4-oxoazetidin-2-yl)phenyl) morpholine-4-carboxamide* ***(2b)***

White powder, yield 73%, mp: 144-146 °C; IR (KBr, cm^-1^): 1643 (CO, urea), 1751 (CO, *β*-lactam); 3456 (NH). ^1^H NMR (250 MHz, DMSO-d_6_) δ (ppm): 3.42 (m, 4H, CH_2_-N), 3.53 (m, 4H, CH_2_-O), 3.67 (s, 3H, OMe), 5.61 (d, *J* 4.6 Hz, 1H, H-4), 5.89 (d, *J* 4.6 Hz, 1H, H-3), 6.88–7.43 (m, 11H, ArH), 8.51 (s, 1H, NH, D_2_O exchange). ^13^C NMR (62.9 MHz, DMSO-d_6_) δ (ppm): 44.3 (CH_2_-N), 55.4 (OMe), 60.5 (C-4), 66.1 (CH_2_-O), 80.8 (C-3), 114.7, 116.1, 118.7, 119.2, 122.3, 125.5, 125.8, 127.9, 128.5, 129.5, 130.1, 140.8, 150.9, 156.2 (aromatic carbons), 155.0 (CO, urea), 161.4 (CO, *β*-lactam); Anal. calcd for C_27_H_25_Cl_2_N_3_O_5_ (542.41): C, 59.79; H, 4.65; N, 7.75%. Found: C, 59.75; H, 4.67; N 7.71%. 

*N-(4-(3-(4-chlorophenoxy)-1-(4-methoxyphenyl)-4-oxoazetidin-2-yl)phenyl) morpholine-4-carboxamide* ***(2c)***

White powder, yield 66%, mp: 158-160 °C; IR (KBr, cm^-1^): 1666 (CO, urea), 1735 (CO, *β*-lactam), 3418 (NH). ^1^H NMR (250 MHz, DMSO-d_6_) δ (ppm): 3.04 (m, 4H, CH_2_-N), 3.33 (m, 4H, CH_2_-O), 3.67 (s, 3H, OMe), 5.44 (d, *J* 4.5 Hz, 1H, H-4), 5.77 (d,* J* 4.5 Hz, 1H, H-3,), 6.81–7.35 (m, 12H, ArH), 8.52 (s, 1H, NH, D_2_O exchange). ^13^C NMR (62.9 MHz, DMSO-d_6_) δ (ppm): 44.1 (CH_2_-N), 55.2 (OMe), 60.5 (C-4), 65.9 (CH_2_-O), 80.5 (C-3), 114.5, 116.7, 118.5, 119.0, 125.4, 125.7, 128.2, 129.0, 130.0, 140.4, 155.2, 155.9 (aromatic carbons), 154.9 (CO, urea), 161.7 (CO, *β*-lactam); Anal. calcd for C_27_H_26_ClN_3_O_5 _(507.97): C, 63.84; H, 5.16; N, 8.27%. Found: C, 63.78; H, 5.15; N, 8.30%. 

*N-(4-(1-(4-methoxyphenyl)-3-(naphthalen-2-yloxy)-4-oxoazetidin-2-yl)phenyl) morpholine-4-carboxamide* ***(2d)***

White powder, yield 95%, mp: 206-208 °C; IR (KBr, cm^−1^): 1658 (CO, urea), 1743 (CO, *β*-lactam), 3425 (NH). ^1^H NMR (250 MHz, DMSO-d_6_) δ (ppm): 3.34 (m, 4H, CH_2_-N), 3.42 (m, 4H, CH_2_-O), 3.67 (s, 3H, OMe), 5.69 (d,* J* 4.5 Hz, 1H, H-4), 5.91 (d, *J* 4.5 Hz, 1H, H-3), 6.88–7.78 (m, 15H, ArH), 8.46 (s, 1H, NH, D_2_O exchange). ^13^C NMR (62.9 MHz, DMSO-d_6_) δ (ppm): 44.0 (CH_2_-N), 55.2 (OMe), 60.7 (C-4), 65.9 (CH_2_-O), 80.5 (C-3), 108.6, 114.5, 117.8, 118.5, 119.0, 124.0, 125.9, 126.5, 126.7, 127.5, 128.2, 128.8, 129.3, 130.1, 133.6, 140.4, 154.8, 155.8 (aromatic carbons), 154.2 (CO, urea), 161.9 (CO, *β*-lactam); Anal. calcd for C_31_H_29_N_3_O_5_ (523.58): C, 71.11; H, 5.58; N, 8.03%. Found: C, 71.15; H, 5.54; N, 8.00%. 

*N-(4-(3-(4-chlorophenoxy)-1-(4-ethoxyphenyl)-4-oxoazetidin-2-yl)phenyl) morpholine-4-carboxamide* ***(2e)***

White powder, yield 69%, mp: 216-218 °C; IR (KBr, cm^−1^): 1666 (CO, urea), 1743 (CO, *β*-lactam), 3425 (NH). ^1^H NMR (250 MHz, DMSO-d_6_) δ (ppm): 1.23 (m, 3H, Me), 3.37 (m, 4H, CH_2_-N), 3.53 (t, 4H, CH_2_-O), 3.89 (q, 2H, OCH_2_), 5.57 (d, *J* 4.6 Hz, 1H, H-4), 5.77 (d, *J* 4.6 Hz, 1H, H-3), 6.81–7.41 (m, 15H, ArH), 8.49 (s, 1H, NH, D_2_O exchange). ^13^C NMR (62.9 MHz, DMSO-d_6_) δ (ppm): 14.5 (Me), 44.1 (CH_2_-N), 60.5 (C-4), 63.1 (OCH_2_), 65.9 (CH_2_-O), 80.5 (C-3), 114.9, 116.7, 118.5, 119.0, 125.4, 125.7, 128.2, 129.0, 129.9, 140.4, 155.2, 155.2 (aromatic carbon), 154.9 (CO, urea), 161.7 (CO, *β*-lactam); Anal. calcd for C_28_H_28_ClN_3_O_5 _(521.99): C, 64.43; H, 5.41; N, 8.05%. Found: C, 64.40; H, 5.42; N, 8.09%. 

*N-(4-(3-(2,4-dichlorophenoxy)-1-(4-ethoxyphenyl)-4-oxoazetidin-2-yl)phenyl) morpholine-4-carboxamide* ***(2f)***

White powder, yield 79%, mp: 192-194 °C; IR (KBr, cm^−1^): 1664 (CO, urea), 1740 (CO, *β*-lactam), 3405 (NH). ^1^H NMR (250 MHz, DMSO-d_6_) δ (ppm): 1.21 (m, 3H, Me), 3.32 (m, 4H, CH_2_-N), 3.53 (m, 4H, CH_2_-O), 3.89 (q, 2H, OCH_2_), 5.60 (d, *J* 4.3 Hz, 1H, H-4), 5.89 (d, *J* 4.3 Hz, 1H, H-3), 6.85–7.42 (m, 11H, ArH), 8.51 (s, 1H, NH, D_2_O exchange). ^13^C NMR (62.9 MHz, DMSO-d_6_) δ (ppm): 14.5 (Me), 44.0 (CH_2_-N), 60.2 (C-4), 63.1 (OCH_2_), 65.9 (CH_2_-O), 80.6 (C-3), 113.1, 114.9, 115.9, 118.5, 118.9, 122.0, 125.3, 127.7, 128.2, 129.2, 129.8, 140.4, 150.9, 155.2 (aromatic carbons), 154.8 (CO, urea), 164.2 (CO, *β*-lactam); Anal. calcd for C_28_H_27_Cl_2_N_3_O_5 _(556.44): C, 60.44; H, 4.89; N, 7.55%. Found: C, 60.49; H, 4.90; N, 7.49%. 

*N-(4-(1-(4-ethoxyphenyl)-4-oxo-3-phenoxyazetidin-2-yl)phenyl) morpholine-4-carboxamide* ***(2g)***

White powder, yield 92%, mp: 206-208 °C; IR (KBr, cm^−1^): 1670 (CO, urea), 1747 (CO, *β*-lactam), 3419 (NH). ^1^H NMR (250 MHz, DMSO-d_6_) δ (ppm): 1.23 (m, 3H, Me), 3.33 (m, 4H, CH_2_-N), 3.52 (t, 4H, CH_2_-O), 3.91 (q, 2H, OCH_2_), 5.57 (d, *J* 4.6 Hz, 1H, H-4), 5.75 (d, *J* 4.6 Hz, 1H, H-3), 6.79–7.36 (m, 13H, ArH), 8.55 (s, 1H, NH, D_2_O exchange). ^13^C NMR (62.9 MHz, DMSO-d_6_) δ (ppm): 14.5 (Me), 44.1 (CH_2_-N), 61.1 (C-4), 63.1 (OCH_2_), 65.9 (CH_2_-O), 80.4 (C-3), 113.5, 114.9, 118.4, 119.0, 121.7, 125.9, 128.2, 129.3, 130.0, 140.4, 155.1, 156.5 (aromatic carbons), 154.9 (CO, urea), 162.1 (CO, *β*-lactam); Anal. calcd for C_28_H_29_N_3_O_5 _(487.55): C, 68.98; H, 6.00; N, 8.62%. Found: C, 69.01; H, 6.03; N, 8.65%. 

*N-(4-(1-(4-ethoxyphenyl)-3-(naphthalen-2-yloxy)-4-oxoazetidin-2-yl)phenyl) morpholine-4-carboxamide* ***(2h)***

White powder, yield 75%, mp: 221-223 °C; IR (KBr, cm^−1^): 1658 (CO, urea), 1743 (CO, *β*-lactam), 3433 (NH). ^1^H NMR (250 MHz, DMSO-d_6_) δ (ppm): 1.23 (m, 3H, Me), 3.33(m, 4H, CH_2_-N), 3.49 (t, 4H, CH_2_-O), 3.89 (q, 2H, OCH_2_), 5.69 (d, *J* 4.6 Hz, 1H, H-4), 5.90 (d, *J* 4.6 Hz, 1H, H-3), 6.87–7.79 (m, 15H, ArH), 8.47 (s, 1H, NH, D_2_O exchange). ^13^C NMR (62.9 MHz, DMSO-d_6_) δ (ppm): 14.5 (Me), 44.0 (CH_2_-N), 60.72 (C-4), 63.1 (OCH_2_), 65.9 (CH_2_-O), 80.5 (C-3), 108.5, 114.9, 117.8, 118.5, 118.9, 124.0, 125.9, 126.5, 126.7, 127.5, 128.2, 128.8, 129.3, 130.0, 133.6, 140.3, 154.8, 155.1 (aromatic carbons), 154.2 (CO, urea), 161.9 (CO, *β*-lactam); Anal. calcd for C_32_H_31_N_3_O_5 _(537.61): C, 71.49; H, 5.81; N, 7.82%. Found: C, 71.56; H, 5.79; N, 7.79%. 

*N-(4-(1-(4-methoxybenzyl)-3-(4-chlorophenoxy)-4-oxoazetidin-2-yl)phenyl) morpholine-4-carboxamide* ***(2i)***

White powder, yield 89%, mp: 186-188 °C; IR (KBr, cm^−1^): 1663 (CO, urea), 1744 (CO, *β*-lactam), 3433 (NH). ^1^H NMR (250 MHz, DMSO-d_6_) δ (ppm): 3.49 (m, 4H, CH_2_-N), 3.62 (m, 4H, CH_2_-O), 3.80 (s, 3H, OMe), 3.88(d, *J* 15.2 Hz, 1H, CH_2_), 4.69 (d, *J *15.2 Hz, 1H, CH_2_), 4.98 (d,* J* 4.3 Hz, 1H, H-4), 5.68 (d, *J* 4.3 Hz, 1H, H-3), 6.85–7.43 (m, 12H, ArH), 8.58 (s, 1H, NH, D_2_O exchange). ^13^C NMR (62.9 MHz, DMSO-d_6_) δ (ppm): 42.8 (CH_2_), 44.1 (CH_2_-N), 55.0 (OMe), 60.2 (C-4), 65.9 (CH_2_-O), 81.3 (C-3), 113.9, 116.6, 118.9, 125.1, 125.9, 127.1, 128.4, 128.9, 129.4, 140.3, 155.2, 158.6 (aromatic carbons), 154.9 (CO, urea), 164.4 (CO, *β*-lactam); Anal. calcd for C_28_H_28_ClN_3_O_5 _(521.99): C, 64.43; H, 5.41; N, 8.05%. Found: C, 64.40; H, 5.37; N, 8.09%. 

*N-(4-(1-(4-chlorophenyl)-4-oxo-3-phenoxyazetidin-2-yl)phenyl) morpholine-4-carboxamide* ***(2j)***

White powder, yield 45%, mp: 235-237 °C; IR (KBr, cm^−1^): 1658 (CO, urea), 1751 (CO, *β*-lactam), 3418 (NH). ^1^H NMR (250 MHz, DMSO-d_6_) δ (ppm): 3.33 (m, 4H, CH_2_-N), 3.52 (m, 4H, CH_2_-O), 5.64 (d, *J* 4.8 Hz, 1H, H-4), 5.81 (d, *J* 4.8 Hz, 1H, H-3), 6.79–7.42 (m, 13H, ArH,), 8.53 (s, 1H, NH, D_2_O exchange). ^13^C NMR (62.9 MHz, DMSO-d_6_) δ (ppm): 44.1 (CH_2_-N), 60.8 (C-4), 65.9 (CH_2_-O), 80.6 (C-3), 114.9, 118.7, 119.1, 121.8, 125.4, 127.9, 128.2, 129.3, 129.3, 135.5, 140.5, 156.4 (aromatic carbons), 154.9 (CO, urea), 162.9 (CO, *β*-lactam); Anal. calcd for C_26_H_24_ClN_3_O_4 _(477.94): C, 65.34; H, 5.06; N, 8.79%. Found: C, 65.28; H, 5.07; N, 8.73%. 

*N-(3-(3-(4-chlorophenoxy)-1-(4-methoxyphenyl)-4-oxoazetidin-2-yl)phenyl) morpholine-4-carboxamide* ***(2k)***

White powder, yield 89%, mp: 114-116 °C; IR (KBr, cm^−1^): 1639 (CO, urea), 1739 (CO, *β*-lactam), 3465 (NH). ^1^H NMR (250 MHz, DMSO-d_6_) δ (ppm): 3.35 (m, 4H, CH_2_-N), 3.53 (m, 4H, CH_2_-O), 3.67 (s, 3H, OMe), 5.56 (d, *J* 4.4 Hz, 1H, H-4), 5.81 (d, *J* 4.4 Hz, 1H, H-3), 6.82–7.82 (m, 12H, ArH), 8.61 (s, 1H, NH, D_2_O exchange). ^13^C NMR (62.9 MHz, DMSO-d_6_) δ (ppm): 44.1 (CH_2_-N), 55.2 (OMe), 60.9 (C-4), 65.9 (CH_2_-O), 80.6 (C-3), 114.5, 116.9, 118.4, 119.5, 121.8, 125.5, 128.1, 129.0, 129.1, 130.1, 133.1, 140.4, 155.3, 155.9 (aromatic carbons), 154.9 (CO, urea), 161.7 (CO, *β*-lactam); Anal. calcd for C_27_H_26_ClN_3_O_5 _(507.97): C, 63.84; H, 5.16; N, 8.27%. Found: C, 63.83; H, 5.19; N, 8.32%. 

*N-(3-(3-(2,4-dichlorophenoxy)-1-(4-methoxyphenyl)-4-oxoazetidin-2-yl)phenyl) morpholine-4- carboxamide* ***(2l)***

White powder, yield 74%, mp: 140-142 °C; IR (KBr, cm^−1^): 1637 (CO, urea), 1767 (CO, *β*-lactam), 3286 (NH). ^1^H NMR (250 MHz, DMSO-d_6_) δ (ppm): 3.34 (m, 4H, CH_2_-N), 3.53 (m, 4H, CH_2_-O), 3.68 (s, 3H, OMe), 5.59 (d, *J* 4.7 Hz, 1H, H-4), 5.92 (d, *J* 4.7 Hz, 1H, H-3), 6.89–7.48 (m, 11H, ArH), 8.53 (s, 1H, NH, D_2_O exchange). ^13^C NMR (62.9 MHz, DMSO-d_6_) δ (ppm): 44.1 (CH_2_-N), 55.2 (OMe), 60.6 (C-4), 65.9 (CH_2_-O), 80.7 (C-3), 114.5, 116.2, 118.4, 118.4, 119.4, 121.7, 122.3, 125.7, 127.7, 128.0, 129.3, 130.0, 132.8, 140.4, 150.8, 156.0 (aromatic carbons), 154.9 (CO, urea), 161.2 (CO, *β*-lactam); Anal. calcd for C_27_H_25_Cl_2_N_3_O_5 _(542.41): C, 59.79; H, 4.65; N, 7.75%. Found: C, 59.81; H, 4.64; N, 7.71%. 

*N-(3-(1-(4-methoxyphenyl)-4-oxo-3-phenoxyazetidin-2-yl)phenyl) morpholine-4-carboxamide* ***(2m)***

White powder, yield 68%, mp: 123-125 °C; IR (KBr, cm^−1^): 1643 (CO, urea), 1743 (CO, *β*-lactam), 3418 (NH). ^1^H NMR (250 MHz, DMSO-d_6_) δ (ppm): 3.33 (m, 4H, CH_2_-N), 3.43 (m, 4H, CH_2_-O) 3.68 (s, 3H, OMe), 5.57 (d, *J* 4.7 Hz, 1H, H-4), 5.79 (d, *J* 4.7 Hz, 1H, H-3), 6.80–7.48 (m, 13H, ArH), 8.63 (s, 1H, NH, D_2_O exchange). ^13^C NMR (62.9 MHz, DMSO-d_6_) δ (ppm): 44.1 (CH_2_-N), 55.2 (OMe), 61.1 (C-4), 65.9 (CH_2_-O), 80.6 (C-3), 114.5, 115.2, 118.4, 118.4, 119.5, 121.8, 121.9, 128.1, 129.3, 130.2, 133.4, 140.4, 155.9, 156.6 (aromatic carbons), 154.9 (CO, urea), 162.2 (CO, *β*-lactam); Anal. calcd for C_27_H_27_N_3_O_5 _(473.52): C, 68.48; H, 5.75; N, 8.87%. Found: C, 68.40; H, 5.79; N, 8.93%. 

*N-(3-(1-(4-methoxyphenyl)-3-(naphthalen-2-yloxy)-4-oxoazetidin-2-yl)phenyl) morpholine-4-carboxamide* ***(2n)***

White powder, yield 63%, mp: 220-222 °C; IR (KBr, cm^−1^): 1674 (CO, urea), 1743 (CO, *β*-lactam), 3441 (NH). ^1^H NMR (250 MHz, DMSO-d_6_) δ (ppm): 3.32 (m, 4H, CH_2_-N) , 3.52 (m, 4H, CH_2_-O) 3.68 (s, 3H, OMe), 5.68 (d, *J* 4.7 Hz, 1H, H-4), 5.95 (d, *J* 4.7 Hz, 1H, H-3), 6.90–7.78 (m, 15H, ArH), 8.51 (s, 1H, NH, D_2_O exchange). ^13^C NMR (62.9 MHz, DMSO-d_6_) δ (ppm): 44.0 (CH_2_-N), 55.2 (OMe), 61.1 (C-4), 65.9 (CH_2_-O), 80.6 (C-3), 108.9, 114.5, 117.9, 118.4, 118.4, 119.4, 121.9, 124.1, 126.5, 126.7, 127.4, 128.1, 128.9, 129.3, 130.2, 133.4, 133.6, 140.3, 154.9, 155.9 (aromatic carbon), 154.3 (CO, urea), 162.0 (CO, *β*-lactam); Anal. calcd for C_31_H_29_N_3_O_5_ (523.58): C, 71.11; H, 5.58; N, 8.03%. Found: C, 71.18; H, 5.55; N, 8.04%. 

*General procedure for antimalarial activity*

The chloroquine resistant *P. falciparum* strain K14 (Southeast Asia) was maintained in type O^+ ^human red blood cells in complete medium supplemented with 10% human serum, at 37 °C, under an atmosphere of 85% N_2_/5% O_2_/5% CO_2 _(26). The cultures were synchronized by sorbitol treatment (27) parasite sensitivity to *β*-lactams was determined using the* in-vitro* isotopic microtest (28). Briefly, the parasites were cultured (synchronized at ring stage containing 0.8% parasitemia and 1.5% hematocrit) in the presence of serial dilutions of *β*-lactam derivatives. 

The parasite growth was assessed by adding 1 µCi of tritiated hypoxanthine with a specific activity of 14.1 Ci/mmol (Perkin-Elmer, Courtaboeuf, France) to each well at time zero. After 48 h of culturing, the plates were freeze-thawed and harvested on filters. The dried filters were moistened in a scintillation liquid mixture (Microscint O, Perkin-Elmer) and the radioactivity incorporated into parasite nucleic acid was counted using a Top Count Microbeta counter (Perkin-Elmer). Drug concentrations inhibiting parasite growth by 50% (IC_50_) were calculated using nonlinear regression analysis of the dose-response curves (Riasmart; Packard, Meridem, USA). The reference antimalarial drug used was chloroquine. The IC_50_ for chloroquine of the K14 strain was 1147.5 ± 31.8 nM.

## Results and Discussion

*Chemistry*

**Scheme 1:**
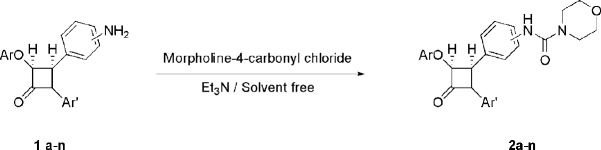
Synthesis of compounds **2a-n**

*β*-lactams bearing the amino group **1a-n** were treated with morpholine-4-carbonyl chloride and triethylamine under solvent free grinding condition to afford the -corresponding *β*-lactams **2a-n **in moderate to high yields (Scheme 1 and Table 1). Using the optimized reaction conditions, *β*-lactams **1a-n** were converted into their corresponding *β*-lactams **2a-n** in moderate-to-excellent yields. These newly synthesized 2-azetidinones have been characterized by spectral data and elemental analyses.

In the IR spectra of compounds **2a-n**, a sharp band in the region at 1735-1767 cm^-1^ is assigned to the stretching of ν (C=O, *b-*lactam). Presence of this band is a critical step in the proof of the *b-*lactam structure of these compounds (29). All of these compounds showed a peak at 1637-1674 cm^-1 ^due to ν (C=O, urea), that is an evidence of the formation of desired products. In addition, the IR spectra of **2a-n** exhibited the –NH stretching between 3286-3465 cm^-1^.

^1^H NMR spectra of the compounds **2a-h **and** 2j-n** displayed H-3 and H-4 protons of *b-*lactam ring between 5.75-5.95 and 5.44-5.69 ppm and **2i** showed H-3 and H-4 protons at 5.67 and 4.98 ppm as doublet. The observed coupling constants (*J* = 4.3-4.7 Hz) for H-4 and (*J* = 4.3-4.7 Hz) for H-3 confirmed the *cis* stereochemistry for all of these compounds (30). **2i** exhibited the benzylic protons at 3.88 and 4.69 ppm as doublets. The spectra of compounds **2a-d**, **2i**, and** 2k-n** also displayed the methoxy proton peak as singlet. The morpholine ring›s protons in all cases appeared as two multiplets. ^1^H NMR spectra of all compounds also displayed the -NH proton (D_2_O exchangeable) as a singlet in the region at 8.46-8.63 ppm. ^13^C NMR spectra of compounds **2a-n** displayed *b-*lactam carbonyl peak at 161.4-164.4 ppm. Urea carbonyl peak appeared in the region at 154.2-155.0 ppm. Carbon of morpholine ring (CH_2_-O) for **2b **was appeared at 66.1 and for the other compounds was appeared at 65.9 ppm. All compounds showed carbons of morpholine ring (CH_2_-N) between 44.0-44.3 ppm.

*Antimicrobial and antibacterial screening (in-vitro)*

All of these newly synthesized *β-*lactams derivatives were subsequently evaluated for their biological activities. First of all, it has been demonstrated that these compounds do not possess significant antimicrobial efficiency against Gram-positive *S. aureus* and Gram-negative bacteria* E. coli* or *P. aeruginosa* and fungi such as *C.albicans* and *C. glabrata* with MICs values greater than 125 µg/mL in all cases. Based on their structural properties, these compounds may not be useful as chelating agents with potential activity. The results of present investigation support the suggested structures of hypothetical antibacterial pharmacophore site. It has been suggested that some functional groups such as azomethine cotaining hetero-aromatics as arm, constitutes the essential pharmacophore site (N --- N) or (N --- O) displayed role of antibacterial activity that may be responsible for the increase of hydrophobic character and liposolubility of molecules. This in turn, enhances activity of the compounds and biological absorbance, so as, all of the synthesized *β*-lactams bearing the amino group **1a-n** and **2a-n** have low antibacterial properties.

A number of important points emerge concerning the electronic and steric factors which have direct impact on bioactivity properties. 

The positive results we have recorded, while encouraging for purposes of new drug design, confirm that very likely most of these compounds could be used as potential antimalarial activity. 

These results prompt several pertinent observations: (i) This type of lactams can furnish an interesting model for studying the interaction of antibiotics with malarial target because the possible charge modification of substituent of pharmacophore groups; (ii) The future flexible pharmacophore site (s) geometric conformation enables us to prepare molecules for multi-therapeutic materials with high selectivity.

*Antimalarial screening (in-vitro)*

Nevertheless, moderate to good antimalarial activities have been obtained against chloroquine resistant *Plasmodium falciparum* K14 strain as outlined in Table 2 with IC_50_ varying from 21 to 50 µM. On the other hand, no significant antimalarial activity was encountered for *β-*lactam derivatives **1a-1n**, all of this suggesting a quite strong influence of the structure of the considered lactam derivative substituted on the anilino group on the mechanism of action involved.

*POM analyses*

For the development of bioactive compounds, the identification of the active structural features is important. Calculations of energetics, atomic charges, minimum energy structures, geometry, and natural bond orbital (NBO) could indicate the electronic density distribution of each atom. These systematic data, regarding the variation of molecular properties, are important for the chemical structure and could therefore provide first insights into the chemical bonding of *b-*Lactam derivatives (LACD) with various targets.

The objective of this study is to investigate the potential pharmacophore sites of LACD species using anti-microbial/ antimalarial screenings dependence on pH and comparison with the calculated molecular properties. To verify these structural features responsible for bioactivity, further Petra/Osiris/Molinspiration (POM) analyses were carried out for calculation of net atomic charges, bond polarity, atomic valence, electron delocalization, and lipophicity.

**Table 1:** Synthesis of *β*-lactams** 2a-n **under solvent free grinding condition

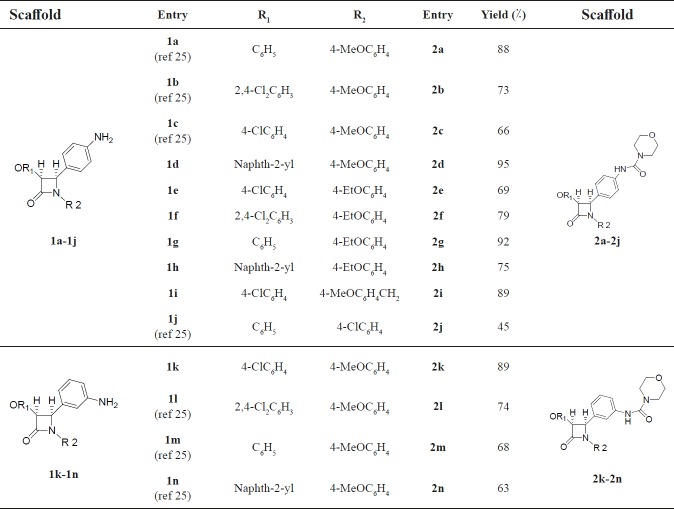

a All products were characterized by IR, ^1^H NMR, ^13^C NMR and elemental analyses.

b Isolated yield after silica gel chromatography.

**Table 2:** Antimalarial activity of the new *β*-lactam derivatives **1a-n **and **2a-n**

**Entry.**	**IC** **50 ** **(µM) ** ***P. falciparum *** **K14**	**Entry.**	**IC** **50 ** **(µM) ** ***P. falciparum *** **K14**
**1a**	>50	**2a**	41
**1b**	50.0	**2b**	21
**1c**	>50	**2c**	30
**1d**	>50	**2d**	41
**1e**	>50	**2e**	>50
**1f**	>50	**2f**	>50
**1g**	>50	**2g**	>50
**1h**	>50	**2h**	>50
**1i**	>50	**2i**	>50
**1j**	41	**2j**	26
**1k**	>50	**2k**	39
**1l**	>50	**2l**	32
**1m**	>50	**2m**	>50
**1n**	>50	**2n**	50

a The standard antimalarial drug (SD) used was chloroquine.

**Table 3:** Osiris calculations of compounds **1a-1n **and **2a-2n**

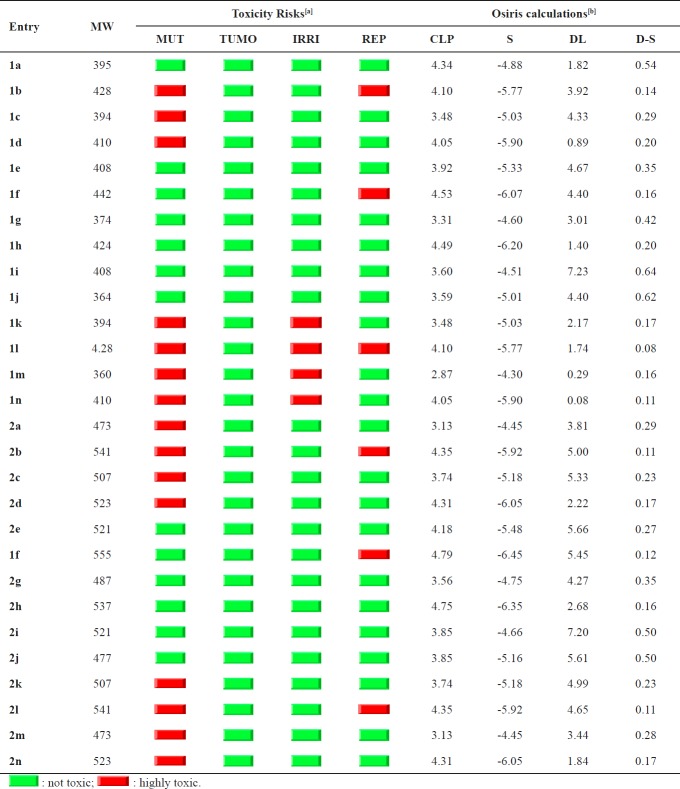

a ^[^^]^MUT: mutagenic; TUMO: tumorigenic; IRRI: irritant; REP: reproductive effective.

b ^[^^]^CLP: cLogP; S: Solubility; DL: Drug-Likness, DS: Drug-Score.

**Table 4:** Molinspiration calculations of compounds **1a-1n **and **2a-2n**

**Entry**	**Molinspiration calculations**				**Drug-likeness**					
	**TPSA**	**NONI**	**NV**	**VOL**	**GPCRL**	**ICM**	**KI**	**NRL**	**PI**	**EI**
**1a**	65	2	0	327	-0.21	-0.26	-0.31	-0.40	-0.25	-0.26
**1b**	65	2	0	354	-0.23	-0.30	-0.28	-0.39	-0.34	-0.30
**1c**	65	2	0	340	-0.21	-0.25	-0.32	-0.40	-0.28	-0.28
**1d**	65	2	0	371	-0.14	-0.20	-0.21	-0.28	-0.18	-0.18
**1** **e**	65	2	0	358	-0.23	-0.26	-0.35	-0.37	-0.29	-0.30
**1f**	65	2	1	371	-0.25	-0.30	-0.32	-0.36	-0.35	-0.32
**1g**	65	2	0	344	-0.24	-0.26	-0.35	-0.37	-0.26	-0.28
**1h**	65	2	1	388	-0.17	-0.21	-0.25	-0.26	-0.20	-0.21
**1i**	65	2	0	358	-0.20	-0.31	-0.26	-0.39	-0.05	-0.27
**1j**	56	2	0	315	-0.19	-0.23	-0.32	-0.40	-0.26	-0.27
**1k**	65	2	0	340	-0.22	-0.26	-0.31	-0.41	-0.29	-0.29
**1l**	65	2	0	354	-0.23	-0.31	-0.28	-0.39	-0.34	-0.32
**1m**	65	2	0	327	-0.22	-0.27	-0.31	-0.40	-0.25	-0.27
**1n**	65	2	0	371	-0.15	-0.21	-0.21	-0.29	-0.19	-0.20
**2a**	77	1	0	429	-0.23	-0.34	-0.34	-0.36	-0.25	-0.31
**2b**	80	1	2	453	-0.17	-0.33	-0.18	-0.33	-0.27	-0.29
**2c**	80	1	1	439	-0.15	-0.27	-0.20	-0.34	-0.23	-0.27
**2d**	80	1	2	469	-0.10	-0.36	-0.16	-0.28	-0.15	-0.23
**2** **e**	80	1	2	456	-0.17	-0.30	-0.23	-0.32	-0.24	-0.28
**1f**	80	1	2	469	-0.19	-0.37	-0.22	-0.32	-0.28	-0.31
**2g**	80	1	0	442	-0.17	-0.27	-0.23	-0.31	-0.21	-0.27
**2h**	80	1	2	486	-0.13	-0.42	-0.22	-0.30	-0.17	-0.28
**2i**	80	1	1	456	-0.16	-0.35	-0.17	-0.32	-0.06	-0.26
**2j**	71	1	0	413	-0.14	-0.21	-0.20	-0.33	-0.21	-0.26
**2k**	80	1	1	439	-0.15	-0.27	-0.20	-0.34	-0.23	-0.27
**2l**	80	1	2	453	-0.17	-0.33	-0.18	-0.33	-0.27	-0.29
**2m**	80	1	0	425	-0.15	-0.25	-0.19	-0.33	-0.20	-0.25
**2n**	80	1	2	469	-0.10	-0.36	-0.16	-0.28	-0.15	-0.23

a TPSA: Total molecular polar surface area; OHN: number of N-H – O interaction; VIOL.: number of violation of ﬁve Lipinsky rules; VOL.: volume.

b GPC: GPCR ligand; ICM: Ion channel modulator; KI: Kinase inhibitor; NRL: Nuclear receptor ligand; PI: Protease inhibitor; EI: Enzyme inhibitor.

**Figure 2:**
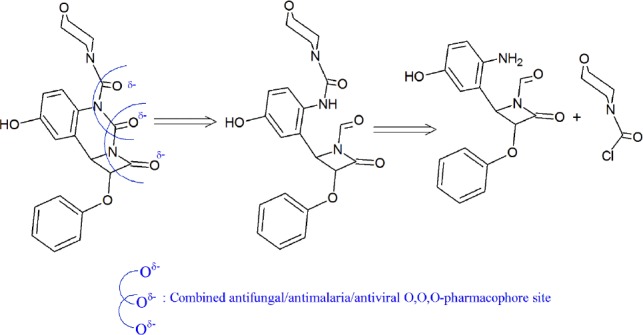
Molecule in perspectives

*Osiris study*

Structure based design is now fairly routine but many potential drugs fail to reach the clinic because of ADME-Tox liabilities. One very important class of enzymes, responsible for many ADMET problems, is the cytochromes P450. Inhibition of these or production of unwanted metabolites can result in many adverse drug reactions. Of the most important program, Osiris is already available online. This is done by using a combined electronic/structure docking procedure and an example will be given here. 

The remarkably well behaved mutagenicity of divers synthetic molecules classified in data base of CELERON Company of Swiss can be used to quantify the role played by various organic groups in promoting or interfering with the way a drug can be associated with DNA.

From the data evaluated in Table 2, it indicates that 11/28 structures are supposed to be non-mutagenic when run through the mutagenicity assessment system and as far as irritating and reproductive effects are concerned, all the 11 compounds are at low risk comparable with their analogesd (**1k-1n**). The log P value of a compound, which is the logarithm of its partition coefﬁcient between *n*-octanol and water, is a well-established measure of the compound’s hydrophilicity. Low hydrophilicity and therefore high log P values may cause poor absorption or permeation. On this basis, all the series of compounds **1a–1n** are having cLogP values under the acceptable criteria should be active. The geometrical parameter and the aqueous solubility of a compound signiﬁcantly affect its absorption, distribution characteristics and bioactivity. Typically, a low solubility goes along with a bad absorption and therefore the general aim is to avoid poorly soluble compounds. 

We have calculated overall drug-score (DS) for the compounds **1a–1n** as shown in Table 3. The DS combines drug-likeness, cLogP, logS, molecular weight, and toxicity risks in one handy value that may be used to judge the compound’s overall potential to qualify for a drug. The reported compounds **2a–2i** showed only 5/28 compounds have good DS (DS = 0.5 or more). That indicates that majority of parameters in drug design have not been realized (Table 3).

*Molinspiration study*

Modern drug discovery is based in large part on high throughput screening of small molecules against macromolecular disease targets requiring that molecular screening libraries contain drug-like or lead-like compounds. We have previously analyzed known standard references (SR) for drug-like and lead-like properties. With this information in hand, we have established a strategy to design specific drug-like or lead-like compounds **1a-1n**. Drug likeness is calculated by the methodology developed by Molinspiration as a virtual screening against various enzymes (Table 4).

The method is very robust and is able to process practically all organic and most organometallic molecules. Molecular Polar Surface Area TPSA is calculated based on the methodology, as a sum of fragment contributions. O- and N- centered polar fragments are considered (31). PSA has been shown to be a very good descriptor characterizing drug absorption, including intestinal absorption, bioavailability, Caco-2 permeability and blood-brain barrier penetration. Prediction results of compounds 1a-1n molecular properties (TPSA, GPCR ligand and ICM) are valued (Table 4).

Oral bioavailability is a desirable property of compounds under investigation in the drug discovery process. Lipinski’s rule-of-five is a simple model to forecast the absorption and intestinal permeability of a compound. In the rule-of-five model, the compounds are considered likely to be well absorbed when they possess these attributes-molecular weight < 500, cLog P < 5, number of H-bond donors < 5, number H-bond acceptors < 10, and number of rotatable bonds < 10. Lipophilicity (log P value) and polar surface area (PSA) values are two important properties for the prediction of oral bioavailability of drug molecules (32). 

The polar surface area (PSA) is calculated from the surface areas that are occupied by oxygen and nitrogen atoms and by hydrogen atoms attached to them. Thus, the PSA is closely related to the hydrogen bonding potential of a compound (32). Molecules with PSA values around of 160 Å or more are expected to exhibit poor intestinal absorption (32). Table 4 shows that all the compounds are under this limit. It has to be kept in mind that log P and PSA values are only two important, although not sufficient criteria for predicting oral absorption of a drug (33). To support this contention, note that all the compounds have 0-1 violation of the Rule-of-five. Two or more violations of the rule of five suggest the probability of problems in bioavailability (34). 

Drug likeness of compounds **1a**-**1n** is tabulated in Table 4. Drug likeness may be defined as a complex balance of various molecular properties and structure features which determine whether particular molecule is similar to the known drugs. These properties, mainly hydrophobicity, electronic distribution, hydrogen bonding characteristics, molecule size and flexibility and also presence of various pharmacophore features influence the behavior of molecule in a living organism, including bioavailability, transport properties, affinity to proteins, reactivity, toxicity, metabolic stability, and many others. Activity of all nine compounds and standard drug were rigorously analyzed under four criteria of known successful drug activity in the areas of GPCR ligand activity, ion channel modulation, kinase inhibition activity, and nuclear receptor ligand activity. The results are shown for all compounds in Table 4 by means of numerical assignment. So, likewise 21/21 compounds have consistent negative values drug score. Therefore, it is readily seen that all the compounds bearing no pharmacophore site are expected to have bad and near ZERO activity to standard drug used based upon these four rigorous criteria (Figure 2).

## Conclusion

In summary, new *β*-lactams bearing morpholine moieties were synthesized by solvent free grinding method in the presence of morpholine-4-carbonyl chloride and Et_3_N. Some remarkable benefits of this method is avoiding the use of solvent, short reaction time, easy purification, moderate to high yield of the products, and also presenting moderate to good antimalarial activities against a *Plasmodium falciparum* K14 resistant strain. Further works are now under current investigation in order to improve the structure of potent more active compounds and elucidate the mechanism of action of these derivatives since the latter remains unclear to date.

The POM analyses of present series **1a**-**1n** support the suggested structures of bioactive containing pharmacophore sites. It has been suggested that some functional groups containing non bonded electron pair, constitutes an essential dipolar fragment which displayed crucial role of biological activity that may be responsible for the bio target/drug interaction. It was shown that the absence of pharmacophore site in the presence of lipophilic groups will not enhance activity of the compounds and biological absorbance, so as, all candidates of the synthesized **1a**-**1n** have modest to poor pharmacological properties.
